# Development of a work systems stress questionnaire to predict job burnout: A mixed methods study based on a macroergonomics approach

**DOI:** 10.1016/j.heliyon.2024.e40226

**Published:** 2024-11-07

**Authors:** Rahman Zare, Reza Kazemi, Alireza Choobineh, Rosanna Cousins, Andrew Smith, Hamidreza Mokarami

**Affiliations:** a Student Research Committee, Department of Occupational Health and Safety Engineering, School of Health, Shiraz University of Medical Sciences, Shiraz, Iran; b Department of Ergonomics, School of Health, Shiraz University of Medical Sciences, Iran; c Research Center for Health Sciences, Shiraz University of Medical Sciences, Iran; d Department of Psychology, Liverpool Hope University, Liverpool, UK; e School of Psychology, Centre for Occupational and Health Psychology, Cardiff University, UK

**Keywords:** Occupational burnout, Work-related stress, Organizational ergonomics, Sociotechnical system, Sequential exploratory design

## Abstract

Job burnout is a stress-related phenomenon that is a significant threat to the health and performance of organizations and employees. Interventions to ameliorate potentials for burnout have been limited by the lack of a comprehensive tool that considers work system stressors. Thus, the aim of this study was to develop a questionnaire for predicting job burnout based on the macroergonomics work system approach. The setting was a petrochemical company in South Iran. In the qualitative phase of this sequential exploratory mixed methods research, 971 meaning codes were extracted from fourteen one-to-one and seven focus group interviews (n = 59). The codes were subject to Directed Content Analysis, which yielded three themes and 15 dimensions, which were used to inform the development of reliable and valid questionnaire. Items for each of the dimensions were sourced from exiting scales. To test the developed Work System Stress Questionnaire (WSSQ) in terms of its ability to predict burnout, a survey which included demographic items, the WSSQ, and the Maslach Burnout Inventory was completed by 359 employees. Hierarchical linear regression modelling of the data indicated that Task Significance, Job Demands, Work-Life Conflict, and Work Schedule predicted Emotional Exhaustion, and altogether explained 58 % variance. Task Significance, Violence and Harassment, Work-Life Conflict, and Job Insecurity predicted the Depersonalization and explained 29 % variance. Decision Latitude, Welfare and Financial Facilities, Task Significance, and Structural Problems predicted Personal Accomplishment and explained 26 % variance. All 15 dimensions were valid (CVI range .73–.90) and reliable (Cronbach's alpha range .71–.93). The results confirm the ability of the WSSQ to explain more variance regarding job burnout than previous studies. In turn, the WSSQ will enable remedial actions to be put into place. It may also be useful for understanding the consequences associated with other organizational ergonomic variables that are related to job stress.

## 1 Introduction

Job burnout is a stress-related phenomenon that is a significant threat to organizational performance through its impact on employees' health and productivity, and its association with low job satisfaction, low organizational commitment, and high turnover [[1], [2], [3]]. Following the early work of Freudenberger [4] and Maslach and Jackson [5], and criticisms that burnout was simply a fashionable diagnosis [6], an authoritative definition of the concept was published by the World Health Organization in 2019 [7]. The 11th edition of the International Classification of Diseases (ICD-11) [7] defines job burnout as an occupational syndrome characterized by feelings of fatigue, and a complete lack of energy; feeling pessimistic and detached about one's job; and decreased efficiency and professional productivity.

Causal factors associated with job burnout have been investigated in many studies using a variety of definitions and methodologies. It can be observed that multiple work-related stress factors are commonly cited as the cause of job burnout, and that although a variety of different job stress models have been employed in the investigations there is currently no theoretical model that is sufficient to provide a comprehensive overview of the stressors associated with job burnout [1]. Similarly, the published tools for identifying job stress are insufficient to predict burnout [1]. From this, it can be realized that a significant gap in the extant burnout literature is a full conceptualization work that recognizes the social and technical aspects involved. Ultimately, the worker is at the center of a work system, and distinguishing the problems and psychological risk factors related to the work system plays an essential role in predicting job stress [8], and in turn, potentials for job burnout.

The macroergonomics perspective can be instructive in moving the literature forward with designing an approach and a tool to predict job burnout. This is the first step towards ameliorating the risks for burnout and its significant impact on both individual and organizational health. Using a macroergonomics approach means that several factors that affect the performance of work systems are considered in predicting burnout, not just psychosocial stressors. The Balance Theory of Job Design [9] uses the macroergonomics approach with a focus on stress reduction to provide the wider perspective on stressors involved in work that is indicated for predicting burnout. According to Balance Theory, a multilevel analysis of the work system is required to characterize stressors which lead to physiological and psychological responses at the individual level. Where there is work with chronic overload and a prolonged stress response, then this will negatively affect human physical and mental health [9]. This can be understood in terms of work system overloads leading to job stress and, if allowed to continue, job burnout in employees [10,11].

The Balance Theory of Job Design [9] describes work organization in terms of five elements which interact to produce a stress load which challenge an individual's biological, psychological and behavioral resources. The five elements of the work system are described as individual (e.g., expertise, impact of aging), task (e.g., workload, job control), tools and technologies (e.g., ergonomic design), physical environment (e.g., noise, heat, layout of workplace), and organization (e.g., shiftwork schedules, reward, recognition). Thus, work system stress is a multidimensional construct that needs to be surveyed for the combined impacts of these five dimensions on employee job burnout. A macroeconomics approach works through maximizing coordination and compatibility of these work systems with their sociotechnical characteristics, leading to synergy to improve several measures of organizational effectiveness, including health, safety, comfort, and productivity [8,12].

To date, the literature does not provide any such comprehensive study on the work system stressors (i.e. personal, organizational, occupational, environmental, and technological stress factors) to afford prediction ofjob burnout of employees. Similarly, there is no published valid and reliable tool to detect work system stressors that have the potential to cause burnout, which is an essential step for enabling purposeful and effective intervention [13]. Thus, the aim of this study was to develop a reliable and valid questionnaire to predict job burnout based on the macroergonomics work system approach.

## 2 Materials and methods

Ethical approval for this research was authorized by the Scientific and Medical Ethics Committee of Shiraz University of Medical Sciences (ethics code: IR. SUMS.REC.1399.1043).

### 2.1 Study design and participants

The study used a sequential exploratory mixed method design. The setting was a petrochemical company in the south of Iran with 922 employees at the time of the study. In the first qualitative phase data was collected from 59 employees using 14 individual semi-structured interviews, and seven focus groups, each with five to eight participants. A purposive sampling method was utilized to recruit participants who were a rich source of information, and were willing to actively participate in our study regarding explaining the conditions of their work system. Decisions regarding who to invite to the study considered criteria of heterogeneity and diversity between participants.

The following quantitative phase used census sampling: all company employees were invited to participate in our study. From the 920 invitations distributed to employees, a total of 402 provided written consent and they were provided with the developed questionnaire, and time to do this at work. Some returned (anonymous) questionnaires (n = 43) had to be eliminated from the database due to missing or distorted data, leaving a total of 359 questionnaires for statistical analyses (39 % of the total population).

**Fig. 1:**
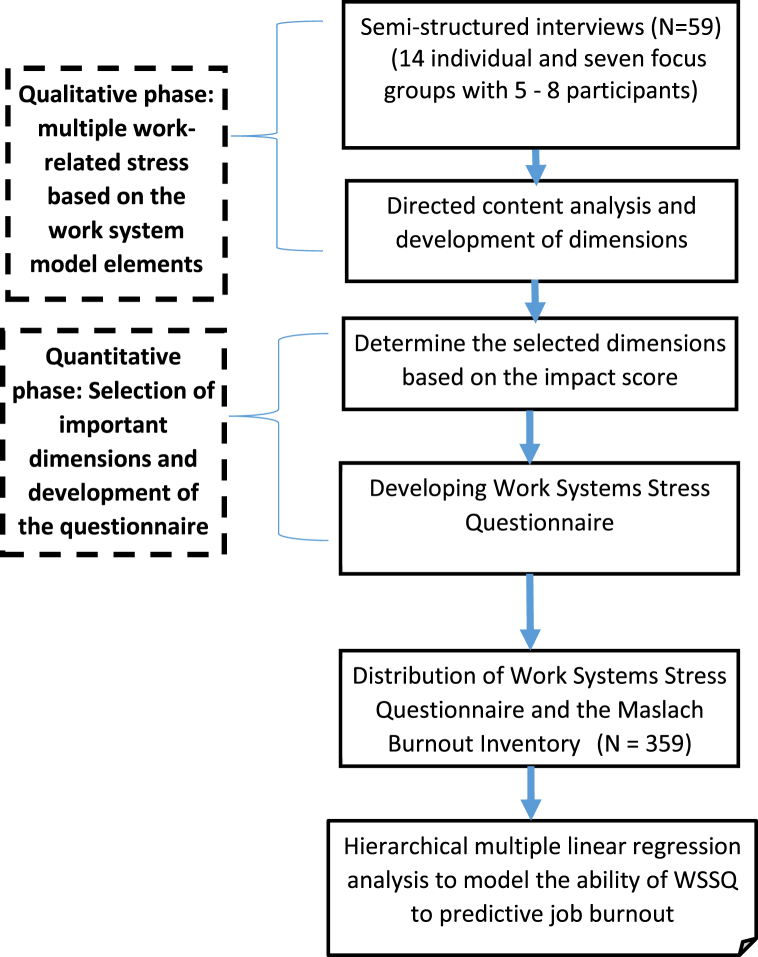
The conceptual study processes.

The conceptual study process is shown in Fig. 1.

### 2.2 Phase 1: qualitative interviews

**Fig. 2:**
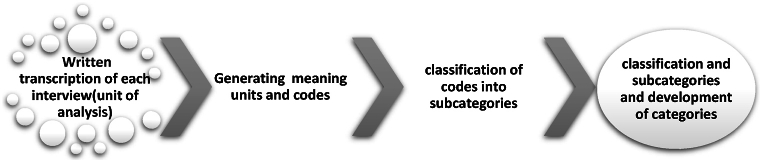
Content analysis process in the qualitative study.

Semi-structured interviews based on the work system model elements were conducted by the first (RZ) and last (HM) authors in 2023. First, information about the study and an invitation to participate was sent to selected employees. Focus groups included employees in similar work systems as a means of collected rich data. With initial consent provided, a mutually convenient appointment was made for those participating in both one-to-one and focus group interviews in a private environment. Information about the research, and its objectives was reiterated at the start of the interviews, and each participant completed a written informed consent form. Interviews lasted between 45 and 80 min, with the focus group interviews being longer. Recordings of the interviews were transcribed by the researchers. Simultaneously with the interview process, the directed content analysis process was performed using the process described by Elo and Kyngäs [14] (see Fig. 2). Theoretical data saturation was confirmed.

### 2.3 Phase 2: quantitative questionnaire development

To ensure the validity of the work system stress dimensions extracted from the qualitative interviews to build a questionnaire, and the deduced categories and sub-categories were provided, along with operational definitions, to a subsample of 200 employees of the consenting participants. These participants were chosen using stratified sampling (proportional to department volume). Their task was to use a checklist to rate the importance (in terms of stress) of these proposed dimensions of the questionnaire using 5-point Likert scale (1 = Not Important; 2 = Slightly Important; 3 = Moderately Important; 4 = Important; 5 = Very Important). The data from the completed checklists (n = 157) was used to calculate an impact score [15]. Dimensions with a mean score higher than four were chosen to design the final questionnaire. For each of the selected dimensions, scales and items of the existing standard psychosocial risk factors questionnaires were utilized to develop a comprehensive Work System Stress Questionnaire. To choose these scales, the following criteria were considered as follows:

1) It should be appropriate and close to the conceptual framework extracted from interviews; 2) The questionnaire should have an English version published in a reputable scientific journal and include published acceptable psychometric properties;
3) The number of items under the desired dimension should be proportional (at least three items);
4) The questionnaire should have generality, as indicated by research in different jobs and workplaces.

Then, the psychometric properties of the developed Work System Stress Questionnaire were investigated using face validity, content validity index (CVI), and internal consistency (Cronbach's alpha coefficient).

#### 2.3.1 Job burnout

To measure the predictive ability of the Work System Stress Questionnaire, the Maslach Burnout Inventory was used [5]. In line with its theoretical underpinning, this questionnaire has three dimensions: Emotional Exhaustion (9 items), Depersonalization (5 items), and Personal Accomplishment (8 items). The 22 items are all scored using a 7-point Likert scale ranging from 0 to 6 and subscale scores were calculated. For emotional exhaustion and depersonalization higher scores show higher burnout, whilst for personal accomplishment low scores are indicative of burnout. The three dimensions of job burnout are non-cumulative [16]. The Persian version used in this study has good reliability: for Emotional Exhaustion α = 0.85, for Depersonalization α = 0.71, and for Personal Accomplishment α = 0.76 [17].

### 2.4 Statistical analysis

MAXQDA-10 software was employed in the qualitative phase to analyze the content and support coding and categorization of the interview and focus group data. SPSS software version 23 (SPSS Inc., Chicago, IL, USA) was used to analyze the quantitative data. The Kolmogorov-Smirnov test was implemented to check the normality of the research data. As all the dependent variables were normally distributed, parametric tests were used for the Inferential analyses.

The effects of sociodemographic variables on the burnout dimensions were investigated using independent t-tests and univariate analyses of variance (ANOVAs). Pearson's correlation coefficient was used to examine the correlations between work system stress variables and the burnout dimensions. Finally, hierarchical multiple linear regression analysis was utilized to develop a predictive model of the three dimensions of job burnout. For modelling, socio-demographics were entered in step 1, and then work system-related stress dimensions were entered in step 2. Variables with p < 0.05 were retained in the final model.

## 3 Results

### 3.1 Phase 1. qualitative study

In total, 971 initial codes were extracted from the fourteen individual interviews and seven focus group interviews (n = 57). The continuous process of integrating similar codes with identical meaning loads produced 276 compacted codes which were put into 41 sub-categories and 21 categories. Finally, the correspondence categories and sub-categories extracted were classified into three themes: organizational factors, job/task factors, and environmental factors (See Table 1).

**Table 1:** Categories, sub-categories and sample quotes from the qualitative content analysis.

Theme	Category	Sub-category	Sample Quotes (translated into English)
Organizational factors	Management support	Lack of management support for employees	… “the company has a slogan ‘the greatest asset of our company is its human resources’ but has significantly lost its impact over time. … it's a real issue we face. Personnel work in a very different situation, and the decline is evident when we look back”. (P6, M, 33 years old)
		Lack of support and value for employees	
	Performance evaluation	Abuse of performance evaluation methods	“Employees frequently comment that their performance evaluations seem subjective. They feel that their performance could be rated higher but believe that evaluations are influenced by their manager's personal preferences. This has become a major concern among staff”. (P3, M, 46 years)
		Biased performance evaluation methods	
	Job promotion	Promotion in job and organization	“Someone with just two-year's experience and no prior knowledge was given a job category of 15. Meanwhile, another individual who spent 14 years at the site and five years in the control room was only given a job category of 14.” (P4, M. 35 years)
	Work schedule	Problems of the work schedule	“It's preferable to start night shifts after a vacation. For the first three or four days, our body's biological clock is disrupted. This affects my family too—for example, my wife has to stay awake with me”. (P11, M, 42 years)
		Inconsistency in work due to the work schedule	
		Work-life conflict due to work schedules	
	Rules and regulation	Lack of adherence to the rules. Inefficient policies and procedures	“There is a system in place, but it operates based on personal preferences”. (P11, M, 42 years)
	Fairness and Justice	Organizational injustice	“You sit here and wonder why things happen the way they do. For example, why do some people get certain services while others, who work just as hard, don't? Why is there a disparity between colleagues?” (P8, M, 33 years)
	Welfare and Financial facilities	Decline of facilities and insufficient compensation	“We used to have regional celebrations that were truly special, with families attending and everyone being warmly welcomed. Now, my wife asks why those celebrations no longer happen. She wonders if this means we have become worthless to the organization since our family seems to be devalued”. (P6, M, 33 years)
		Lack of facilities in the field	
		Challenges with (car) transportation services	
	Structural problems	Frequent changes in structure of the organization	“This constant change in management has exhausted everyone. Each time a new manager comes in, we have to adapt to their style and methods, whether they're effective or not. In the past 5 years, we've had four different managers”. (P1, F, 39 years)
		Management instability	
		Poor organizational governance structure	
	Violence and harassment	Managers' lack of trust/suspicions of employees	“At work, I often feel disheartened and end up doing things my own way. When I encounter trouble and turn to my manager, I feel judged and stigmatized”. (P1, F, 39 years)
		Employees slandering each other	
	Information sharing	Communication deficits and lack of transparency in the provision of information	“There's no transparency in the organization. Whenever someone is transferred, although HR could explain the situation, information gets passed down secretly through the layers of the organization, leading to a lack of openness”. (P4, M, 35 years)
	Management characteristics	Stress-generating management style	“The management system seems to thrive on creating anxiety and stress. During meetings, we are frequently asked why we aren't stressed, and why we seem so comfortable and carefree”. (P5, M, 42 years)
		Non-participatory management	
		Management capability	
	Social-supportive climate	Social climate of the organization	“The rumors, essentially gossip, are things like 'today this person had this conversation with that one and did something wrong,' or 'today that person was behaving like this'”. (P7, M, 43 years)
		Manager-employee relationships	
		Social-supportive climate of colleagues	
	Participating in decision making	Lack of employee involvement in decision making	“When a company wants to implement a policy, it does whatever it wants. It might call and ask the personnel for their thoughts, but in the end, they usually proceed with their own plans”. (P7, M, 43 years)
Job (Task) factors	Job demands	High physical workload	“Work in our unit must be performed with precision and urgency”. (P2, M, 43 years) “I've been out of the house for nearly 13 h, before the overtime. Our regular working hours are too long, and due to a shortage of employees, we're required to work overtime”. (P10, F, 31 years old)
		Time pressures	
		High cognitive demands	
	Decision latitude	Responsibility without authority	“Our job involves making decisions about the future of employees and people. The title sounds impressive, but in reality, it carries no authority”. (P1, F, 39 years)
	Task significance	Meaning of Work	“I'm an expert. I give my opinion, but after I have done so, it gets lost in the chain. No one acknowledges who gave the opinion, what it was about, or the reasoning behind it. This lack of recognition demotivates people. After 15 years, I feel my work has become almost meaningless”. (P1, F, 39 years)
	Job security	Job insecurity	“If you ask anyone in the company, from the lowest to the highest level, they will say job security is a concern. It doesn't matter if it's me, my supervisor, or our manager—we all face this issue”. (P10, F, 31 years)
Environmental factors	Exposure to harmful workplace	Exposure to toxic chemicals, hot and humid work environments	“The air is hot, we're exposed to chemicals, and we work in noisy place”. (P4, M, 35 years)
	Designing the workplace	Layout and space of workplace	“I mentioned to my colleagues that the neighboring petrochemical company have bought comfortable chairs for their staff, and they are very satisfied with them. One of our colleagues checked our chairs and noticed some issues. For example, some people's feet don't touch the ground, and thus their legs hang down, causing fatigue”. (P11, M, 42 years)
	Safety hazards	Accidental work environment	“When a valve breaks down or a line gets damaged, our unit has to handle it. We must consider the risks involved, including the potential for explosions”. (P8, M, 33 years_
	Living in industrial Areas	Difficult living conditions	“… no social interactions in the city we lived in. Compared to the people who lived there, we had no social life”. (P8, M, 33 years)

### 3.2 Phase 2. quantitative questionnaire development

According to the impact scores, 15 work system dimensions were recognized as stressful by the participants. These were: exposure to harm in the workplace; (high) job demands, (insufficient) fairness and justice, (meaningless) rules and regulation, (poor) welfare and financial facilities, (unclear) task significance, (inadequate) management support, structural problems, violence and harassment, (processes for) job promotion, job insecurity, work-life conflict, work schedules, (low) decision latitude (control), and (inadequate) information sharing.

The final multiple work system stress questionnaire contained these 15 dimensions and 60 items. The dimensions of this tool include the Demands subscale in the 10.13039/501100000869HSE Management Standards Stress Indicator Tool [13]; the Management Support subscale in the Job Content Questionnaire [18]; the Job Promotion subscale in the Effort-Reward Imbalance Questionnaire [19]; the Meaning of Work and Influence at Work in the Copenhagen Psychological Questionnaire [20,21]; the Perceived Job Insecurity Scale [22]; the Conflict between Work-Life and Private Life dimension in the Danish Psychosocial Work Environment Questionnaire [23]; the Violence and Harassment subscale in the Negative Acts Questionnaire-Revised [24], and the Environmental Hazards of Work, Fairness and Justice, Rules and Regulations, Welfare and Financial Facilities, Structural Problems, Work Schedule, and Information Sharing tools developed by Mokarami [25]. The results of the validity (mean CVI score) and internal consistency (Cronbach's alpha) of the questionnaire dimensions according to the findings of this study (N = 359) are listed in Table 2.

**Table 2:** Validity (mean CVI score) and reliability (Cronbach's alpha) of WSSQ dimensions (N = 359).

Dimension	Number of items	Cronbach's alpha	CVI Total
1. Harmful Workplace	3	.71	.73
2. Job Demands	8	.86	.77
3. Fairness and Justice	5	.87	.82
4. Rules and Regulations	4	.92	.83
5. Welfare and Financial Facilities	4	.78	.88
6. Task Significance	3	.87	.90
7. Management Support	4	.88	.83
8. Structural Problems	4	.74	.63
9. Violence and Harassment	4	.88	.91
10. Job Promotion	3	.72	.93
11. Job Security	4	.80	.85
12. Work-life Conflict	3	.92	.89
13. Work Schedule	4	.93	.72
14. Decision Latitude	4	.79	.80
15. Information Sharing	3	.90	.81

The mean (SD) age and job tenure of employees were 38.5 (4.6) years and 11.7 (4.2) years. The sample comprised >90 % men, and 90 % had a bachelor's degree or higher. Table 3 reports the socio-demographic characteristics of the participants.

**Table 3:** Sociodemographic characteristics of participants (N = 359).

Characteristics		n	%
**Gender**	Male	334	93 %
	Female	25	7 %
**Educational status**	Diploma	8	2.2 %
	Associate	32	8.9 %
	Bachelor's degree	218	60.7 %
	Postgraduate MSc and PhD	101	28.1 %
**Marital Status**	Single	42	11.7 %
	Married	317	88.3 %
**Residence**	Resident	214	59.6 %
	Non-resident	145	40.4 %
**Work-schedule**	Consistent daytime work	70	19.5 %
	Flying daytime work^a^	24	6.7 %
	Intermittent daytime work^b^	116	32.3 %
	Rotating shift work	149	41.5 %

a At weekends, commute from work to home, and return, by airplane.

b 14 days on, 14 days off, working 12-h days.

Table 4 explains differences in the job burnout dimension according to socio-demographic characteristics. The results indicated significant differences in Emotional Exhaustion according to gender, marital status and age. No significant influence of these or other characteristics were observed in Personal Accomplishment or Depersonalization.

**Table 4:** Mean (SD) and tests of difference in sociodemographic characteristics and job burnout dimensions (N = 359).

		Emotional Exhaustion	Depersonalization	Personal Accomplishment
**Gender**	Men	(10.31)13.92	(4.65) 4.16	(8.22)30.58
	Women	21.84 (10.41)	5.36 (5.84)	30.58 (8.22)
	***P-value***	**< 0.001**	.224	.548
**Marital Status**	Single	19.88 (10.46)	4.88 (5.39)	29.21 (8.8)
	Married	13.76 (10.31)	4.16 (4.66)	30.68 (8.8)
	***P-value***	**< 0.001**	.356	.275
**Residence**	Resident	14.58 (10.4)	4.27 (4.59)	30.38 (7.7)
	Non-resident	14.31 (10.68)	4.21 (4.98)	30.69 (8.81)
	***P-value***	.809	.918	.727
**Age (years)**	≤35	17.31 (10.28)	4.8 (4.77)	29.78 (7.56)
	36–40	13.64 (10.37)	3.95 (4.66)	31.08 (8.23)
	40–43	14.48 (10.17)	4.83 (4.33)	29.69 (7.2)
	≥43	12.21 (10.97)	3.56 (5.4)	30.81 (9.92)
	***P-value***	**0.021**	.292	.54
**Education**	Diploma	9.5 (8.67)	3 (2.78)	32.5 (10.54)
	Technician	14.97 (11.64)	5.22 (5.66)	30.19 (10.43)
	BSc	14.21 (10.46)	3.99 (4.65)	30.14 (7.9)
	MSc/PhD	10.35 (10.35)	4.59 (4.74)	31.25 (7.76)
	***P-value***	.457	.379	.615
**Job tenure (years)**	≤5	14.41 (10.02)	4.05 (4.85)	31.27 (8.02)
	5–10	16.07 (10.41)	4.30 (4.99)	29.73 (8.26)
	10–15	13.08 (10.27)	3.75 (4.19)	31.11 (7.77)
	≥15	16.10 (11.24)	5.63 (5.50)	29.41 (9.08)
	***P-value***	.093	.062	.36
**Work-schedule**	Permanent daytime work	15.69 (10.88)	3.91 (4.76)	30.13 (7.15)
	Flying daytime work	18.17 (9.71)	3.58 (4.42)	30.54 (7)
	Intermittent daytime work	14.62 (10.31)	4.68 (4.64)	31.03 (7.55)
	Rotating shift work	13.19 (10.47)	4.17 (4.88)	30.27 (9.21)
	***P-value***	.105	.611	.859

A significant correlation was founded between all work system stress dimensions with the three dimensions of job burnout (see Table 5). Regarding Emotional Exhaustion, the highest correlation was observed with Work-life Conflict (r = .649). For Personal Accomplishment, the strongest relationship was observed with the Task Significance dimension (r = −.402). For Depersonalization, the highest correlation was observed in the dimension of Violence and Harassment (r = .453).

**Table 5:** Correlations between study variables: three job burnout dimensions and fifteen work system-related stress dimensions (N = 359).

	Mean	SD	1	2	3	4	5	6	7	8	9	10	11	12	13	14	15	16	17
1. Emotional Exhaustion	14.47	10.50	–																
2. Personal Accomplishment	17.49	8.16	−.462^b^	–															
3. Depersonalization	4.25	4.74	.672^b^	−.378^b^	–														
4. Harmful Workplace	10.51	2.35	.303^b^	−.150^b^	.170^a^	–													
5.5. Job Demands	22.45	5.88	.606^b^	−.263^b^	.364^b^	.348^b^	–												
6. Fairness and Justice	9.01	3.04	.459^b^	−.296^b^	.360^b^	.353^b^	.491^b^	–											
7. Rules and Regulations	20.45	6.29	.512^b^	−.380^b^	.393^b^	.319^b^	.527^b^	.780^b^	–										
8. Welfare and Financial Facilities	12.56	3.82	.333^b^	−.285^b^	.231^b^	.313^b^	.383^b^	.397^b^	.448^b^	–									
9. Task Significance	5.88	2.75	.540^b^	−.402^b^	.426^b^	.261^b^	.397^b^	.469^b^	.544^b^	.432^b^	–								
10. Management Support	10.34	3.86	.376^b^	−.260^b^	.375^b^	.260^b^	.388^b^	.451^b^	.471^b^	.320^b^	.491^b^	–							
11. Structural Problems	13.30	3.22	.399^b^	−.192^b^	.276^b^	.167^a^	.496^b^	.452^b^	.617^b^	.464^b^	.396^b^	.445^b^	–						
12. Violence and Harassment	8.56	3.88	.528^b^	−.354^b^	.453^b^	.351^b^	.524^b^	.532^b^	.626^b^	.352^b^	.550^b^	.565^b^	.462^b^	–					
13. Job Promotion	8.23	2.48	.362^b^	−.345^b^	.324^b^	.200^b^	.288^b^	.414^b^	.453^b^	.237^b^	.429^b^	.389^b^	.264^b^	.475^b^	–				
14. Job Security	8.56	3.88	.345^b^	−.293^b^	.185^b^	.225^b^	.337^b^	.398^b^	.476^b^	.341^b^	.386^b^	.314^b^	.388^b^	.465^b^	.468^b^	–			
15. Work-life Conflict	8.31	3.25	.649^b^	−.341^b^	.448^b^	.320^b^	.657^b^	.450^b^	.519^b^	.389^b^	.447^b^	.381^b^	.431^b^	.585^b^	.337^b^	.361^b^	–		
16. Work Schedule	10.48	4.64	.562^b^	−.351^b^	.366^b^	.272^b^	.478^b^	.410^b^	.493^b^	.339^b^	.419^b^	.358^b^	.353^b^	.460^b^	.340^b^	.335^b^	.626^b^	–	
17. Decision Latitude	12.34	3.21	.276^b^	−.347^b^	.179^a^	.230^b^	.238^b^	.250^b^	.309^b^	.091	.306^b^	.234^b^	.152^a^	.329^b^	.345^b^	.293^b^	.186^b^	.208^b^	–
18. Information Sharing	8.18	3.07	.482^b^	−.341^b^	.360^b^	.235^b^	.554^b^	.579^b^	.694^b^	.405^b^	.480^b^	.461^b^	.558^b^	.637^b^	.400^b^	.457^b^	.577^b^	.498^b^	.331^b^

a p < 0.05.

b p < 0.01.

The results of regression modelling indicated that Job Demands, Task Significance, Work-Life Conflict, and Work Schedule had a significant effect on Emotional Exhaustion. The strongest predictor of this dimension of job burnout was Work-Life Conflict (β = .919, p-value <.001). The adjusted variance of the final model revealed that these variables predicted 55 % of Emotional Exhaustion (as listed in Table 6).

**Table 6:** Summary of the hierarchical linear regression analysis of Emotional Exhaustion (N = 359).

*Predictor*^*s*^	Step 1			Step 2		
	*B*	*β*	*SE*	*B*	*β*	*SE*
Gender (female)	6.702^b^	.163	2.137	*___*	*ns*	*___*
Marital status (single)	−4.390^a^	1.802	1.802	*___*	*ns*	*___*
Job Demands				.393^c^	.220	.093
Task Significance				.859^c^	.225	.183
Work-Life Conflict				.919^c^	.284	.184
Work Schedule				.333^b^	.147	.110

**R**^**2**^	**0.071**			**0.578**		
**Adjusted R**^**2**^	**0.061**			**0.554**		

^a^ only significant predictors are reported.

a p < 0.05.

b p < 0.01.

c p < 0.001.

Regarding Depersonalization, the variables Task Significance, Violence and Harassment, Job Insecurity and Work-Life Conflict were directly and significantly related. Among the variables studied, the strongest predictor was Task Significance (β = .295, p-value <.01). The modified variance of the final model indicated that the predictor variables explained 29 % of Depersonalization (as reported in Table 7).

**Table 7:** Summary of the hierarchical linear regression analysis of Depersonalization (N = 359).

*Predictor*^*s*^	Step 1			Step 2		
	*B*	*β*	*SE*	*B*	*β*	*SE*
Gender (female)	.113^a^	.127	.047	*___*	*ns*	*___*
Task Significance				.295^b^	.171	.104
Violence and Harassment				.188^a^	.154	.087
Job Insecurity				.147^a^	.115	.073
Work-Life Conflict				.290^b^	.198	.104

**R**^**2**^		**0.016**			**0.323**	
**Adjusted R**^**2**^		**0.013**			**0.291**	

^a^ only significant predictors are reported.

a p < 0.05.

b p < 0.01.

In the dimension of Personal Accomplishment, the variables Welfare and Financial Facilities, Task Significance, Structural Problems, and Decision Latitude were inversely and significantly related. Among the variables studied, the strongest predictor of this domain was Decision Latitude (β = −.532, p-value = <.001). The adjusted variance of the final model showed that the predictor variables predicted 26 % of personal accomplishment (see Table 8).

**Table 8:** Summary of the hierarchical linear regression analysis of Personal Accomplishment (N = 359).

*Predictor*^*s*^	Step 2		
	*B*	*β*	*SE*
Welfare and Financial Facilities	−.282∗	−.132	.121
Task Significance	−.443∗	−.149	.182
Structural Problems	−.354∗	−.140	.164
Decision Latitude	−.532∗∗∗	−.210	.131

**R**^**2**^		**0.292**	
**Adjusted R**^**2**^		**0.261**	

∗p < 0.05; ∗∗p < 0.01; ∗∗∗p < 0.001.

^a^ only significant predictors are reported.

## 4 Discussion

The aim of this sequential exploratory mixed methods study was to develop a tool to predict job burnout based on the macroergonomic work systems approach of the Balance Theory of Job Design [9]. The findings confirmed that all the dimensions of the developed Work System Stress Questionnaire (WSSQ) were significantly associated with the three dimensions of the Maslach Burnout Inventory [5], a commonly used research tool to measure burnout. Specifically, the results of the final model of the hierarchical regression analysis indicated that WSSQ variables Job Demands, Task Significance, Work-Life Conflict, Work Schedule, Violence and Harassment, Job Security, Welfare and Financial Facilities, Organizational Structure and Decision Latitude are all involved in job burnout. This study adds to the literature a reliable and valid tool – the WSSQ – that can be used in organizations to successfully indicate work system conditions that are conducive to employee burnout. These findings confirm the value of the macroergonomic approach used in this study through the evidence we have provided of work systems variables involved in employee burnout in addition to those that have previously been reported. We recommend that risk assessments for burnout use our more comprehensive tool, and similarly, these variables should feature in interventions to tackle job burnout.

The methodology used was strong. In the qualitative phase the experienced interviewers used the Work System Model that underpins Balance Theory [9] in their interview guide, and to direct analysis of the large amount of rich data collected. In line with the mixed methods design used, the findings of the qualitative phase were then integrated into the questionnaire development phase. The reliability and validity of dimensions of the WSSQ were then assessed using a large sample of full-time employees. The results indicated high validity and internal consistency, as well as significantly predicting burnout in this large sample of industrial workers. The inferential multiple linear regression analyses showed that the WSSQ was able to predict 58 % of the variance of Emotional Exhaustion, 29 % of the variance of Depersonalization, and 26 % of the variance for Personal Accomplishment. Up to now, few studies have achieved this strength of prediction of job burnout [26].

The hierarchical linear regression analyses found Task Significance in the predictive model for all three dimensions of job burnout. There are different definitions of Task Significance, yet most of them mention that in addition to the person needing to experience a sense of personal significance from their work, they also want an objective understanding of the significance and value of what they do at the organizational level [27]. The correlation matrix of the fifteen subscales of the SWWQ also provides some credence to previous considerations that if a person's Job Security is constantly threatened, and there are Violence and Harassment in the organization alongside low Management Support then an employee is likely to conclude that their work is not socially meaningful or valuable in the organization. This provides a strong pointer for the value of an employee's Task Significance for ameliorating potentials of job stress and job burnout.

Besides Task Significance, Work-Life Conflict, Work Schedule and Job Demands were also found to be strong predictors of Emotional Exhaustion. One of the possible reasons for this finding was that most employees were working at some distance from their family home. In almost all the interviews, employees mentioned the high number of hours, and issues with their work schedules and the relatively remote petrochemical industries sites, which altogether caused problems for supportive communications with their family. This is important in the Petrochemicals industry, and many others where the conditions of the region, and distance from family add to existing job stress factors and increase potentials for burnout. In line with the assertion that a challenging Work Schedule is stressful and a predictor of burnout, a study among veterinary technicians [28] also reported that an employee's work schedule was one of the strongest factors associated with job burnout in all three dimensions.

Regarding our findings of the Job Demands subscale predicting burnout, our findings provide the replication requested by Ravalier and colleagues [29] who examined the utility of the Management Standards Indicator Tool [13] in assessing the risk of burnout using the MBI at least for this subscale. Interestingly, Job Demands was the only one of the seven work-related stressors included in their regression analyses to significantly predict Emotional Exhaustion, explaining 30 % of the variance. A similar figure was found in this study, but additional variance was found in our study using the more comprehensive WSSQ from Task Significance, Work-Life Conflict, Work Schedule which amounted to 58 % variance. This comparative analysis provides strong support for the value of using the WSSQ as a comprehensive predictor of burnout.

Various studies have shown that work overload, both qualitative (mental) and quantitative (physical), drain people's capacity to fulfil their job demands. Moreover, when overload is a chronic and stable condition and not an occasional emergency, the person has little opportunity to rest, recover, and restore balance [30,31]. Ultimately, high Job Demands from long working hours and high mental and physical workload, impacts capacity to operate effectively and there are thresholds beyond which they cannot recover. To prevent burnout, this stressor should be carefully managed.

This discussion has also indicated that there are close relations and interactions among predictor variables. Job Demands, Work Schedule and Work-life Conflict, such that changes in each of these factors can affect the other. For instance, when a person has a large overload and is repeatedly forced to work overtime outside of time (high job demand), it is not possible for him to take leave on his own (inadequate schedule). As a result, it would cause some family problems (work-life conflict). Nevertheless, at this point, it is not reasonable to suggest that there is a primary predictor among these variables, and without evidence to the contrary, these variables, and indeed each of the fifteen different aspects of the work system stress model represented in the questionnaire is relevant to understanding Emotional Exhaustion, in particular. Finally, regarding significant risk factors for Emotional Exhaustion, in this study employees who do not have a sense of Meaningful Work reported higher scores in Emotional Exhaustion. Similar results have been reported in a study investigating job burnout process among oncologists [26]. This should be noted as important in future intervention studies.

In terms of the Depersonalization dimension, significant predictors were Task Significance, Job insecurity, Work-Life Conflict and Violence and Harassment. Rasmussen and colleagues [26] also found task significance to be a significant predictor of depersonalization among their sample of oncologists. A small study among disability support workers conducted multiple regression modelling to predict burnout in this cohort of this dimension also found Work-Life Conflict, and gender significantly predicted depersonalization score (n = 98), explaining 4.4 % and 3.6 % of variance individually [32]. In contrast to this study, the sample was largely female. The higher risk for females is indicated in both studies. The gender difference may also underpin the other differences in outcome, but it is also likely to be the type of work. Other significant predictors of burnout in that work system included Role Ambiguity (7.8 %), Work Hours (5.8 %) Client Challenging Behavior (4.4 %), and lack of Supervisor Support (4.4 %). As discussed above, these variables are likely to be correlated, and the presentation of unique contribution to the variance to Depersonalization as done in this publication clouds the overall picture regarding how much variance is explained. Nevertheless, this study too also indicates the need to take a much broader analysis of the work system that has been taken in most other studies examining predictors of burnout. Alongside this point, it is worthwhile mentioning here, that one of the reasons that different organizations predict distinct types of job burnout justifications in different organizations can be interpreted as that each organization has its own unique work system. Hence, employees may encounter different stress factors that can predict different dimensions of job burnout (here, depersonalization). Besides, according to the conducted interviews, we believe that the lampoon culture in the organization we studied, as well as the type of common dialogue in the masculine Petrochemical industry, which can be called verbal violence, has been a risk factor leading to depersonalization in this study, which could be likened to the Client Challenging Behavior studied in the Disability Support Worker Study.

In the Personal Accomplishment dimension of burnout, the final model showed Decision Latitude (control) was the strong predictor. In addition, Task Significance, Welfare and Financial Facilities and Structural Problems were important significant predictors that require attention. Decision Latitude was also found to have a strong stimulating effect on Personal Accomplishment in a study conducted among nurses which modelled burnout as a mediator of patient care [33]. Regarding Task Significance, interestingly, in the Burnout in Oncologists study previously mentioned, Task Significance was only important regarding its relationship with Emotional Exhaustion and Depersonalization. Rasmussen et al. [26] observed that increasing the amount of positive feedback that these professionals received could be a good way to decrease job burnout. In this way, we believe when people see their presence in the organization as useful and receive feedback on this usefulness, they will be less stressed.

In the case of Structural Problems, an examination of these items in the WSSQ indicates that the organization's bureaucracy and organizational chart are mentioned. Interestingly, in a study to identify unknown related factors that led to job burnout in nurses, bureaucracy was one of the factors suggested by experts [34]. Here, based on what we know of the existing organizational structure from our interviews with employees, there have been constant changes in managers and organizational rules, high levels of management and organizational hierarchy, and a disproportionate organizational chart from lack of promotion over time and lack of manpower, which culminated in feelings of low personal accomplishment.

Alongside this was the contribution of a sense of (poor) Welfare and Financial Facilities to burnout associated with (poor) Personal Accomplishment. Similarly, Bahadori et al. [35] described that among the factors that decrease employee job burnout was reduced working hours and improved facilities and conveniences.

### 4.1 Limitations and suggestions for future research

Among the limitations of the paper are that self-reporting tools were employed, so participants may have been biased in providing their answers. This is difficult to overcome as most of the variables are subjective. Sampling in the quantitative phase was performed by the available census in which the participants were people who participated voluntarily, and anonymity in all aspects of the validation of the questionnaire should have provide reassurance that participant's responses were not going to reflect on their work performance evaluations. Whilst an attempt was made to provide a fully comprehensive Work Systems Stress Questionnaire, in the *individual* terms of the work system, personality traits were not included to predict job burnout. It can be also observed that the dimension *tools and technologies* did not contribute to job burnout at all in this study. This may be due to the condition of the organization being well aware of the need for best practice in this safety critical industry, but there are also concerns that there may be impacts for job security from declaring issues related to this theme in the organization. This study was conducted only in one (petrochemical) industry, and as already discussed gender was found to be a predictor of burnout in this and other burnout studies, so this variable should always be considered in stress and burnout studies. Future studies should replicate the methodology to predict job burnout as well as to assess the similarities between stress factors of work systems in different organizations and industries – and in due course, to develop interventions. It should be noted that most of the stress factors identified in the qualitative phase were in the phase of organizational factors, thus we suggest that a mixed method approach is the best approach for extending this investigation on the organizational factors of the industrial system to predict job burnout. Finally, the WSSQ developed in this study should be utilized to confirm its psychometric properties as well as to predict burnout and other stress consequences in other industries.

## 5 Conclusion

This study has developed a Work System Stress Questionnaire that can effectively predict job burnout. For the first time, this study has provided a reliable and valid tool to support identification of work system stressors, and follow-up purposeful remedial actions to be put into place to protect organizational and employee health and performance. The sequential explanatory mixed methods approach indicated relevant dimensions in the initial qualitative phase, which could then be integrated into a relevant tool for risk assessment purposes, that could be quantitatively tested. Consideration of relevant literature indicates that whilst there may be differences in predictors according to an organization's work system, the general stressors identified in this study are not peculiar to the industry in which the study took place. In the present study, Task Significance was biggest predictor of job burnout, which should be focused on in the development of future intervention and control measures to prevent job burnout. Future studies may confirm the psychometric properties of the WSSQ and its ability to predict burnout. It may also be useful for understanding the consequences associated with other organizational ergonomic variables such as job dissatisfaction and turnover intentions that are also related to job stress.

## CRediT authorship contribution statement

**Rahman Zare:** Writing – review & editing, Writing – original draft, Methodology, Data curation. **Reza Kazemi:** Writing – original draft, Resources, Project administration, Methodology, Funding acquisition, Conceptualization. **Alireza Choobineh:** Writing – original draft, Resources, Funding acquisition, Conceptualization. **Rosanna Cousins:** Writing – review & editing, Methodology. **Andrew Smith:** Writing – original draft, Validation, Methodology, Investigation. **Hamidreza Mokarami:** Writing – review & editing, Writing – original draft, Validation, Software, Project administration, Methodology, Investigation, Funding acquisition, Conceptualization.

## Data availability

The anonymized data used and/or analyzed during the current study are available from the corresponding author upon reasonable request.

## Declaration of Competing Interest

The authors declare that they have no known competing financial interests or personal relationships that could have appeared to influence the work reported in this paper.
