# Supplementary material for: Development of a work systems stress questionnaire to predict job burnout: A mixed methods study based on a macroergonomics approach
Source: Heliyon. 2024 Nov 7;10(23):e40226. doi: 10.1016/j.heliyon.2024.e40226 (PMC11626067; doi:10.1016/j.heliyon.2024.e40226)
Supplement: Multimedia component 1 [file mmc1.pdf]

Burnout Self-Test Maslach Burnout Inventory (MBI)

The Maslach Burnout Inventory (MBI) is the most commonly used tool to self-assess whether you might be at risk of burnout. To determine the risk of burnout, the MBI explores three components: exhaustion, depersonalisation and personal achievement. While this tool may be useful, it must not be used as a clinical diagnostic technique, regardless of the results.

The objective is simply to make you aware that anyone may be at risk of burnout.

For each question, indicate the score that corresponds to your response relevant to that time. Add up your score for each section and compare your results with the scoring results interpretation at the bottom of this document.

Questions	Never	A Few Times per Year	Once a Month	A Few Times per Month	Once a Week	A Few Times per Week	Every Day
**Section A**	**0**	**1**	**2**	**3**	**4**	**5**	**6**
I am feel emotionally drained by my work.							
Working with people all daylong requires a great deal of effort.							
I feel like my work is breaking me down.							
I feel frustrated by my work.							
I feel that I work too hard at my job.							
It stresses me too much to work in direct contact with people.							
I feel like I am at the end of my tether.							
**Subscores**							

**Section A Total Score**	

Questions	Never	A Few Times per Year	Once a Month	A Few Times per Month	Once a Week	A Few Times per Week	Every Day
**Section B**	**0**	**1**	**2**	**3**	**4**	**5**	**6**
I feel I deal with colleagues or clients impersonally, as if they were objects.							
I feel tired when I get up in the morning and have to face another day at work.							
I have the impression that my colleagues or clients make me responsible for some of their problems.							
I am at the end of my patience at the end of my work day.							
I really don't care about what happens to some of my colleagues/ clients.							
I am more insensitive to people I was working with.							
I am afraid that the job was making me uncaring.							
**Subscores**							

**Section B Total Score**	

Burnout Self-Test Maslach Burnout Inventory (MBI) | © 2021 monkeypuzzletraining.co.uk

Questions	Never	A Few Times per Year	Once a Month	A Few Times per Month	Once a Week	A Few Times per Week	Every Day
**Section C**	**0**	**1**	**2**	**3**	**4**	**5**	**6**
I have accomplished many worthwhile things in the job.							
I feel full of energy.							
I can easily understand what my colleagues or clients feel.							
I look after my colleagues' or clients' problems very effectively.							
In my work, I handle emotional problems very calmly.							
Through my work, I feel that I have a positive influence on people.							
I am easily able to create a relaxed atmosphere with my colleagues or clients.							
I feel refreshed when I have been close to my colleagues or clients at work.							
**Subscores**							

**Section C Total Score**	

How to interpret your scores : VIEW ONLINE

or visit: *monkeypuzzletraining.co.uk/burnout-inventory-results*

Adapted from:
C. Maslach, S.E. Jackson, M.P. Leiter (Eds.), Maslach Burnout Inventory manual (3rd ed.), Consulting Psychologists Press (1996)
